# Case report of scrub typhus complicated by hypokalemia and multiple organ dysfunction syndrome

**DOI:** 10.1590/1516-3180.2023.0142.R1.08122023

**Published:** 2024-03-11

**Authors:** Li Chen, Yi Deng, Peiying Huang, Sisi Lei, Shuling Liu, Weitao Lin, Zhishang Li, Jing Zeng, Miaochun Huang, Qiuping Huang, Qihua Wu, Haobo Zhang, Bojun Chen

**Affiliations:** I MD. Associate Chief Physician, Associate Professor, Emergency Department of Guangdong Provincial Hospital of Traditional Chinese Medicine, Guangzhou City, Guangdong Province, China; Member, Guangdong Provincial Key Laboratory of Research on Emergency in Traditional Chinese Medicine, Clinical Research Team for Prevention and Treatment of Cardiac Emergencies with Traditional Chinese Medicine, Guangzhou City, Guangdong Province, China.; II MD. Physician, The Second Clinical Medical School of Guangzhou University of Chinese Medicine, Guangzhou City, Guangdong Province, China; Member, Guangdong Provincial Key Laboratory of Research on Emergency in Traditional Chinese Medicine, Clinical Research Team for Prevention and Treatment of Cardiac Emergencies with Traditional Chinese Medicine, Guangzhou City, Guangdong Province, China.; III MD. MSc. Physician, Doctoral Student, The Second Clinical Medical School of Guangzhou University of Chinese Medicine, Guangzhou City, Guangdong Province, China; Member, Guangdong Provincial Key Laboratory of Research on Emergency in Traditional Chinese Medicine, Clinical Research Team for Prevention and Treatment of Cardiac Emergencies with Traditional Chinese Medicine, Guangzhou City, Guangdong Province, China.; IV MD, MSc. Doctoral Student, The Second Clinical Medical School of Guangzhou University of Chinese Medicine, Guangzhou City, Guangdong Province, China; Member, Guangdong Provincial Key Laboratory of Research on Emergency in Traditional Chinese Medicine, Clinical Research Team for Prevention and Treatment of Cardiac Emergencies with Traditional Chinese Medicine, Guangzhou City, Guangdong Province, China.; V MD, MSc. Physician, Doctoral Student, Emergency Department of Guangdong Provincial Hospital of Traditional Chinese Medicine, Guangzhou City, Guangdong Province, China; Member, Guangdong Provincial Key Laboratory of Research on Emergency in Traditional Chinese Medicine, Clinical Research Team for Prevention and Treatment of Cardiac Emergencies with Traditional Chinese Medicine, Guangzhou City, Guangdong Province, China.; VI MD, MSc. Attending physician, Emergency Department of Guangdong Provincial Hospital of Traditional Chinese Medicine, Guangzhou City, Guangdong Province, China; Member, Guangdong Provincial Key Laboratory of Research on Emergency in Traditional Chinese Medicine, Clinical Research Team for Prevention and Treatment of Cardiac Emergencies with Traditional Chinese Medicine, Guangzhou City, Guangdong Province, China.; VII MSc. Associate Chief Physician, Lecturer, Emergency Department of Guangdong Provincial Hospital of Traditional Chinese Medicine, Guangzhou City, Guangdong Province, China; Member, Guangdong Provincial Key Laboratory of Research on Emergency in Traditional Chinese Medicine, Clinical Research Team for Prevention and Treatment of Cardiac Emergencies with Traditional Chinese Medicine, Guangzhou City, Guangdong Province, China.; VIII MD. Chief Physician, MD, Emergency Department of Guangdong Provincial Hospital of Traditional Chinese Medicine, Guangzhou City, Guangdong Province, China; Member, Guangdong Provincial Key Laboratory of Research on Emergency in Traditional Chinese Medicine, Clinical Research Team for Prevention and Treatment of Cardiac Emergencies with Traditional Chinese Medicine, Guangzhou City, Guangdong Province, China.; IX Nurse, Nurse-in-charge, Emergency Department of Guangdong Provincial Hospital of Traditional Chinese Medicine, Guangzhou City, Guangdong Province, China; Member, Guangdong Provincial Key Laboratory of Research on Emergency in Traditional Chinese Medicine, Clinical Research Team for Prevention and Treatment of Cardiac Emergencies with Traditional Chinese Medicine, Guangzhou City, Guangdong Province, China.; X Nurse, Associate Chief Nurse, Emergency Department of Guangdong Provincial Hospital of Traditional Chinese Medicine, Guangzhou City, Guangdong Province, China; Member, Guangdong Provincial Key Laboratory of Research on Emergency in Traditional Chinese Medicine, Clinical Research Team for Prevention and Treatment of Cardiac Emergencies with Traditional Chinese Medicine, Guangzhou City, Guangdong Province, China.; XI MD, MSc. Physician, Master of Medicine, Emergency Department of Guangdong Provincial Hospital of Traditional Chinese Medicine, Guangzhou City, Guangdong Province, China; Member, Guangdong Provincial Key Laboratory of Research on Emergency in Traditional Chinese Medicine, Clinical Research Team for Prevention and Treatment of Cardiac Emergencies with Traditional Chinese Medicine, Guangzhou City, Guangdong Province, China.; XII MD, MSc. Physician, Emergency Department of Guangdong Provincial Hospital of Traditional Chinese Medicine, Guangzhou City, Guangdong Province, China; Member, Guangdong Provincial Key Laboratory of Research on Emergency in Traditional Chinese Medicine, Clinical Research Team for Prevention and Treatment of Cardiac Emergencies with Traditional Chinese Medicine, Guangzhou City, Guangdong Province, China.; XIII MD, MSc. Chief Physician, Professor, Emergency Department of Guangdong Provincial Hospital of Traditional Chinese Medicine, Guangzhou City, Guangdong Province, China; Team leader, Guangdong Provincial Key Laboratory of Research on Emergency in Traditional Chinese Medicine, Clinical Research Team for Prevention and Treatment of Cardiac Emergencies with Traditional Chinese Medicine, Guangzhou City, Guangdong Province, China.

**Keywords:** Scrub typhus, Multiple organ failure, Hypokalemia, Multiple organ dysfunction syndrome, Hypokalemia, Tsutsugamushi disease

## Abstract

**CONTEXT:**

Scrub typhus, caused by *Orientia tsutsugamushi*, has a wide range of clinical manifestations, including meningoencephalitis, acute renal failure, pneumonitis, myocarditis, and septic shock. However, there are no documented cases of scrub typhus with hypokalemia. In this report, we present a case of scrub typhus with hypokalemia and multiple organ failure syndrome, highlighting the importance of electrolyte imbalance in patients with scrub typhus.

**CASE REPORT:**

A 59-year-old woman presented to the emergency department with abdominal pain that had been present for 1 day. On admission, the physical examination and laboratory test results indicated that the patient had renal, liver, and circulatory failure, and hypokalemia. She developed meningitis and disseminated intravascular coagulation during hospitalization. She recovered with appropriate management, and was discharged on day 17.

**CONCLUSION:**

This report highlights the potential for atypical presentations of scrub typhus, including a previously undocumented association with hypokalemia. Although the contribution of hypokalemia to the patient’s clinical course remains uncertain, this case underscores the importance of considering electrolyte imbalance in the management of patients with scrub typhus. Further research is warranted to better understand the relationship between scrub typhus and electrolyte imbalance.

## INTRODUCTION

Scrub typhus, caused by *Orientia tsutsugamushi*, is a significant public health concern in the Asia-Pacific region.^1^ Patients may present with various signs of infection 5 to 14 days after a bite from an infected vector.^1^ Severe scrub typhus can result in a range of complications, including jaundice, acute renal failure, pneumonitis, acute respiratory distress syndrome, myocarditis, septic shock, meningoencephalitis, pericarditis, and disseminated intravascular coagulation (DIC).^2^ Despite the effectiveness of tetracycline, delayed therapy and complications are associated with a 30% mortality rate.^3^

In clinical practice, hypokalemia is a common electrolyte imbalance is a typical clinical manifestation, characterized by weakness.^4^ Severe hypokalemia can lead to life-threatening complications such as malignant arrhythmia and respiratory muscle paralysis.^4^ However, to our knowledge, the occurrence of hypokalemia in the context of scrub typhus and its association with multiple organ dysfunction syndrome has not been previously reported. Herein, we present a case of scrub typhus complicated by hypokalemia and multiple organ dysfunction syndrome.

## CASE REPORT

This study was approved by the Institutional Review Board of the Guangdong Provincial Hospital of Chinese Medicine on May 22, 2023 (G2023-13). Written informed consent was obtained from the patient for the publication of this case report and the accompanying images.

The patient, a 59-year-old woman with a history of hypertension presented to the emergency department of a traditional Chinese medicine hospital in Guangdong Province on June 26, 2022 (day 0) with mild abdominal pain, which had been present for 1 day. On admission, her vital signs included a blood pressure of 75/53 mmHg, a heart rate of 85 beats per minute, a body temperature of 36.6°C, and a respiratory rate of 22 breaths per minute. Laboratory test results revealed a platelet count of 71×10^9^ platelets/L and a white blood cell count of 8.92×10^9^ cells/L. The levels of C-reactive protein (239.10 mg/)L, pro-calcitonin (3.97 ng/mL), and blood lactic acid (4.96 mmol/L) were elevated. Liver function tests showed elevated levels of alanine aminotransferase (ALT) (178 U/L), aspartate aminotransferase (AST) (197 U/L), total bilirubin (23.4 μmol/L, and direct bilirubin at 21.3 μmol /L, and decreased levels of total protein (60.0 g/L), and albumin (33.4 g/L). Electrolyte imbalances were also noted, with decreased blood potassium and sodium levels of 2.62 mmol/L and 128 mmol/L, respectively. Creatinine was elevated at 272 μmol/L, and urea was 10.40 mmol/L. Coagulation parameters showed a prolonged prothrombin time of 14.3 s, an elevated activated partial thromboplastin time of 36.8 s, an international normalized ratio of 1.26, and an elevated D-dimer level of 12.64 mg/L. Computed tomography of the chest and abdomen revealed a renal calculus on the left side. Based on the clinical history, laboratory test results, and imaging findings, preliminary diagnoses of septic shock and multiple organ dysfunction syndrome (MODS) involving the liver, kidney, circulation, blood, acute liver failure, acute kidney failure, hypertension, and electrolyte disorders (hypokalemia and hyponatremia) were made. The patient was admitted to the Emergency Intensive Care Unit for further management.

**Figure 1:**
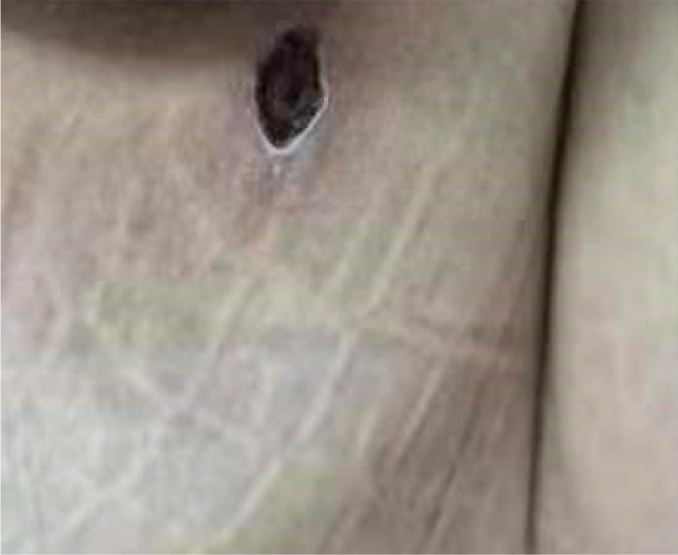
Eschar on the left buttock on day 0.

On physical examination, the patient’s body was found to be covered with small purple skin lesions, and an eschar was present on her left buttock (Figure 1). Given the strong suspicion of scrub typhus, a blood sample was sent for metagenomic next-generation sequencing (mNGS) and quantitative polymerase chain reaction (qPCR) to confirm the diagnosis. Due to worsening hypoxemia and oliguria on day 1, ventilator support and continuous renal replacement therapy were initiated. Antibiotic treatment was modified to doxycycline 100 mg twice daily, considering the patient’s severe condition with MODS and suspected scrub typhus. Supportive measures, including platelet transfusion, liver protection, acid inhibition, and maintenance of homeostasis, were also provided.

**Figure 2:**
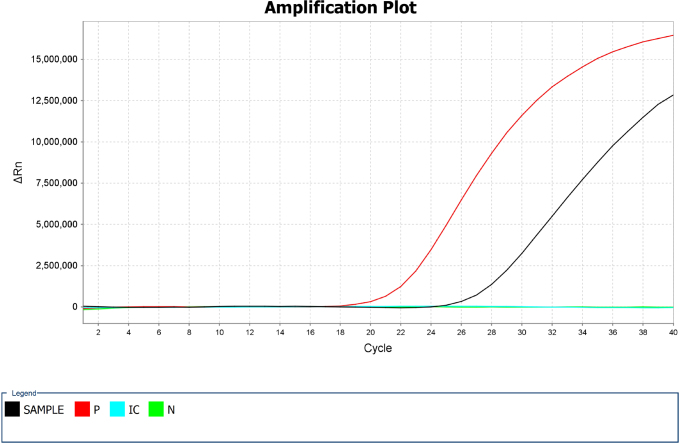
The quantitative polymerase chain reaction (qPCR) test results: The black line shows the amplification of the *Orientia tsutsugamushi,* DNA in the test sample obtained from the patient’s plasma; the red line shows the positive control sample; and the green line shows the negative control sample.

The patient’s condition deteriorated on day 2, with worsening of her level of consciousness, disappearance of physiological reflexes, limb convulsions, swelling of the face and limbs, and worsening ecchymosis in the lower limbs. Laboratory test results showed a decreasing platelet count, an increasing white blood cell count, and abnormal liver function tests with decreasing ALT levels and increasing AST, total bilirubin, direct bilirubin, total protein, and D-dimer levels. The mNGS and qPCR results (Figure 2) confirmed the presence of *O. tsutsugamushi*, suggesting that scrub typhus was the underlying cause.

**Figure 3:**
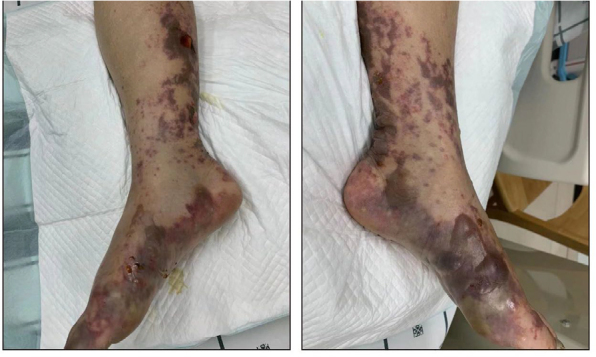
Blisters and ecchymoses on the patient’s lower legs on day 8.

The patient received platelet transfusions and appropriate management, and by day 8, she had started to recover with an improved level of consciousness (Glasgow Coma Scale: E4VTM5). She developed blisters and worsening ecchymoses on the lower legs, scattered bruises, and minor skin lesions all over her body (Figure 3). On day 17, she was transferred to the Rehabilitation Department for further rehabilitation and was discharged 2 days later.

## DISCUSSION

The patient was diagnosed with scrub typhus and experienced multiple complications including renal failure, liver failure, circulatory failure, pneumonia, encephalitis, and DIC. Hypokalemia, was present on admission. The prevalence of hypokalemia in patients with scrub typhus varies in different research reports, ranging from 10% in South India^5^ to 22.9% in South Korea^6^; however, to our knowledge, hypokalemia has not previously been reported in patients in China. The exact cause of hypokalemia in patients with scrub typhus is not well understood; however, several factors could potentially contribute to its development.

First, the patient had a history of hypertension and was taking blood pressure medication; however, specific details of the medication were not available. Some of the diuretics commonly prescribed to manage hypertension can cause hypokalemia by increasing the excretion of potassium in the urine. This could explain the hypokalemia observed in this patient.

Second, the patient had a history of mild abdominal pain and reduced appetite, which could have resulted in a decreased intake of potassium-rich foods. Additionally, the patient received fluid replacement therapy to address shock, which potentially resulting in fluid dilution and subsequently contributing to the development of hypokalemia.

Third, the renal tubular acidosis and the effects of scrub typhus on ion channels that may have disrupted the serum potassium balance. The patient’s serum potassium levels recovered, so no further investigations were conducted to determine the cause of the hypokalemia.

Hypokalemia can cause weakness and other symptoms, and can be life-threatening if left untreated.^7^ Hypokalemia has also been reported in other infectious diseases, including COVID-19).^4^

In summary, although the exact cause of hypokalemia in this patient with scrub typhus remains unclear, it is likely multifactorial and may be related to factors such as fluid balance, medication use, renal tubular acidosis, and disease mechanisms. Further research and investigations are warranted to better understand the pathophysiology of hypokalemia in patients with scrub typhus and to guide appropriate management strategies. This report highlights the importance of monitoring and managing electrolyte abnormalities in patients with scrub typhus and other critical conditions.

## References

[1] Xu G, Walker DH, Jupiter D, Melby PC, Arcari CM (2017). A review of the global epidemiology of scrub typhus. PLoS Negl Trop Dis..

[2] Rajapakse S, Weeratunga P, Sivayoganathan S, Fernando SD (2017). Clinical manifestations of scrub typhus. Trans R Soc Trop Med Hyg..

[3] Rajapakse S, Rodrigo C, Fernando D (2012). Scrub typhus: Pathophysiology, clinical manifestations and prognosis. Asian Pac J Trop Med..

[4] Alfano G, Ferrari A, Fontana F (2021). Hypokalemia in patients with COVID-19. Clin Exp Nephrol..

[5] Grover R, Mehalingam V (2021). Acute kidney injury and electrolyte abnormalities in patients with scrub typhus admitted to a tertiary care hospital in southern india. J Family Med Prim Care..

[6] Park SW, Ha NY, Ryu B (2015). Urbanization of scrub typhus disease in South Korea. PLoS Negl Trop Dis..

[7] Rehman FU, Omair SF, Memon F (2020). Electrolyte imbalance at admission does not predict the length of stay or mortality in dengue-infected patients. Cureus..

